# Wahrnehmungen zum Umgang mit Opioiden: Fokus COVID-19: Eine Umfrage unter Anästhesist:innen über die Fachgesellschaften DGAI/BDA

**DOI:** 10.1007/s00101-022-01101-2

**Published:** 2022-03-16

**Authors:** Vera Peuckmann-Post, Christoph Wiese, András Keszei, Roman Rolke, Frank Elsner

**Affiliations:** 1 Klinik für Palliativmedizin, Medizinische Fakultät, Uniklinik RWTH Aachen, RWTH Aachen University, Aachen, Deutschland; 2 Klinik für Anästhesiologie, Medizinische Fakultät, Uniklinik RWTH Aachen, RWTH Aachen University, Aachen, Deutschland; 3 Klinik für Anästhesiologie und Intensivmedizin, Stiftung Herzogin Elisabeth Hospital, Braunschweig, Deutschland; 4 Center for Translational & Clinical Research Aachen, Medizinische Fakultät, Uniklinik RWTH Aachen, RWTH Aachen University, Aachen, Deutschland

**Keywords:** Morphin, SARS-CoV-2, Symptomkontrolle, Palliativmedizin, Dyspnoe, Morphin, SARS-CoV-2, Symptom management, Dyspnoea, Palliative care

## Abstract

**Hintergrund:**

Opioide gehören zum Klinikalltag in Anästhesiologie, Intensivmedizin und Palliativmedizin. Hinsichtlich der Behandlung von Dyspnoe mit Opioiden finden sich in Leitlinien jedoch unterschiedliche Gewichtungen. Dies kann zu Unsicherheiten bezüglich Indikationsstellung und ethischer Implikationen im Umgang mit Opioiden – auch bei COVID-19 – führen.

**Ziel der Arbeit:**

Erfassung der Wahrnehmung bezüglich Umgang mit Morphin/Opioiden (M/O) zur Symptomkontrolle *inner*- und *außerhalb* der Palliativmedizin, auch bei COVID-19-Erkrankten.

**Material und Methoden:**

Mittels SurveyMonkey® (Momentive Inc., San Mateo, CA, USA) wurden Mitglieder der Deutschen Gesellschaft für Anästhesiologie (DGAI) und des Berufsverbands Deutscher Anästhesisten (BDA) im Oktober 2020 anonymisiert nach ihrer eigenen Wahrnehmung zum Umgang mit M/O zur Symptomkontrolle befragt.

**Ergebnisse und Diskussion:**

Von *N* = 1365 teilnehmenden Anästhesist:innen beschrieben 88 % den Umgang mit M/O *innerhalb* der Palliativmedizin als „sicher und vertraut“ bzw. 85 % als „klar geregelt“, während dies für die Bereiche *außerhalb *der Palliativmedizin deutlich seltener angegeben wurde (77 %/63 %). Bei der Betreuung COVID-19-Erkrankter wurde der Umgang mit M/O *außerhalb* der Palliativmedizin noch seltener als „sicher und vertraut“ (40 %) oder „klar geregelt“ (29 %) wahrgenommen. Dyspnoe (95 %/75 %), Erleichterung des Sterbeprozesses (84 %/51 %), Unruhe (59 %/27 %) und Angst/Panik (61 %/33 %) wurden häufiger *innerhalb* als *außerhalb* der Palliativmedizin als allgemeine Indikationen genannt. Von den Befragten wünschten sich 85 % die Einbindung eines palliativmedizinischen Konsilteams.

**Fazit:**

Anästhesist:innen nahmen deutliche Unsicherheiten im Umgang mit M/O wahr, insbesondere *außerhalb *der Palliativmedizin. Einheitliche, interdisziplinäre Leitlinien zur Symptomkontrolle etwa bei Dyspnoe, mehr Lehre und die Einbindung eines palliativmedizinischen Konsilteams sollten zukünftig intensiver bedacht werden.

## Abstract (en)

**Background:**

Opioids are part of the daily routine in anesthesiology and palliative care; however, treatment of dyspnea with opioids is presented heterogeneously in guidelines. This may result in an uncertainty concerning opioid indications and ethical concerns, especially when caring for COVID-19 patients.

**Objective:**

We aimed to examine the perception of anesthesiologists concerning the handling of morphine as the reference opioid (subsequently termed M/O) for symptom control within and outside of a palliative care setting, including care for COVID-19 patients.

**Material and methods:**

Members of the German Society of Anesthesiology and Intensive Care Medicine (DGAI) and the Professional Association of German Anesthesiologists (BDA) received an anonymized online questionnaire (Survey Monkey®; Momentive Inc., San Mateo, CA, USA) in October 2020, containing questions regarding their perception of symptom management with M/O in general, and in particular concerning COVID-19 patients. Participants were asked to rate their perception within and outside a palliative care setting.

**Results and discussion:**

A total of 1365 anesthesiologists participated; 46% women. Most anesthesiologists were 41–60 years old (58%), worked in a hospital setting (78%), in the operating theatre (63%) and in intensive care units (49%). The majority (57%) reported > 20 years of professional experience (52%) and partial involvement in palliative care (57%). Perception of M/O handling was mostly “certain and confident” (88%) and “clearly regulated” (85%) within a palliative care setting but rated substantially lower for outside palliative care (77%/63%). When caring for COVID-19 patients, handling of M/O was even less often rated “certain and confident” (40%) or “clearly regulated” (29%) outside palliative care. Dyspnea (95%/75%), relief of the dying process (84%/51%), agitation (59%/27%) and anxiety/panic (61%/33%) were more frequently rated as general indications for morphine within versus outside palliative care. The majority of anesthesiologists disputed that M/O is given with the intention to hasten death within (87%) and outside (93%) palliative care. Highest difference in route of administration was reported for the subcutaneous administration of M/O within (76%) versus outside (33%) palliative care, followed by the intravenous route (57%/79%), while oral (66/62%) and transdermal (48%/39%) administration were reported to be used comparatively frequently. Most participants (85%) wanted more frequent involvement of palliative care consultation teams but also more team conferences (75%), supervision (72%), and training on opioid management (69%).

**Conclusion:**

Anesthesiologists perceived considerable uncertainty in using M/O for nonpalliative care medical settings. Highest uncertainty was seen for the care of patients with COVID-19. The prevalent use of the subcutaneous route for M/O application in palliative care can serve as inspiration for areas outside palliative care as well. Uniform interdisciplinary guidelines for symptom control including dyspnea, education and involvement of a palliative care consultation team should be more considered in the future.

## Hintergrund und Fragestellung

Opioide lindern effizient Symptome wie Dyspnoe [17, 24]. Der sichere Umgang mit Opioiden ist daher bedeutend für die individuelle Symptomkontrolle. Empfehlungen hierzu finden in Leitlinien inner- und außerhalb der Palliativmedizin jedoch unterschiedliche Gewichtung [22, 23, 31, 33].

Trifft die mit Dyspnoe einhergehende Coronavirus Erkrankung 2019 (COVID-19) wie ein „Tsunami“ [29] auf das Gesundheitssystem, so könnten vorbestehende Unsicherheiten im Umgang mit Opioiden eine adäquate Patient:innenversorgung gefährden. Daher untersuchten wir Wahrnehmungen von Anästhesist:innen zum Umgang mit Morphin/Opioiden (M/O) *inner*- und *außerhalb* der Palliativmedizin, allgemein und bei Patient:innen mit COVID-19.

### Material und Methoden

Auf Grundlage von Einzel- und Fokusgruppeninterviews mit Ärzt:innen und Pflegenden *innerhalb* wie *außerhalb* der Palliativmedizin (nicht publiziert) sowie der internationalen Literatur identifizierten wir häufige Fragestellungen und Assoziationen zum Umgang mit Opioiden in der Symptomkontrolle. Über das gemeinsame Sekretariat der Deutschen Gesellschaft für Anästhesiologie und Intensivmedizin (DGAI) sowie des Berufsverbandes der Anästhesisten (BDA) wurde per E‑Mail jeweils am 13.10.2020 und 23.10.2020 (Erinnerung) ein Link zu der anonymisierten Online-Umfrage (SurveyMonkey®; Momentive Inc., San Mateo, CA, USA) zugesandt.

Im Fragebogen informierten wir über Initiator:innen, Zeitangabe zur Durchführung und Anonymisierung. Die Eingabe erfolgte manuell, und es war möglich, einzelne Fragen unbeantwortet zu überspringen sowie im Freitextfeld „Weitere Anmerkungen“ Kommentare zu hinterlassen. Die Teilnahme war nur einmalig möglich (IP-Adressen-Registrierung durch SurveyMonkey®). Für den Fall, dass Teilnehmende die Umfrage mehrfach erhalten haben sollten, forderten wir auf, an der Umfrage nur einmal teilzunehmen.

Der Fragebogen beinhaltete soziodemografische Daten und Fragen zur Wahrnehmung der klinischen Anwendung von Opioiden allgemein und speziell bei COVID-19-Erkrankten. Die zuständige Ethikkommission des Uniklinikums Aachen genehmigte die Durchführung der Studie (EK 303/20).

Wir formulierten im Fragebogen: „*Wir sind an Ihren eigenen Erfahrungen interessiert, aber auch daran, wie Sie Kolleginnen und Kollegen anderer Fachrichtungen im Umgang mit Opioiden zur Symptomlinderung wahrnehmen. […] ‚Morphin‘ soll dabei exemplarisch für die Gruppe der Opioide genannt werden.“ *Wir erklärten weiter, dass in dieser Untersuchung bei den Fragen „*zwischen dem palliativmedizinischen Bereich (z.* *B. Palliativstation, ambulanter Palliativdienst) und der Anwendung in anderen Fachkliniken (innere Medizin, Chirurgie, Orthopädie/Unfallchirurgie, Neurologie, Intensivstationen etc.)“ *unterschieden werde solle. Diese Unterscheidung bezeichnen wir nachfolgend als „*innerhalb* der Palliativmedizin“ und „*außerhalb* der Palliativmedizin“.

Zur Erfassung der Einschätzungen setzten wir eine 6‑stufige Likert-Skala ein (von „stimme gar nicht zu“ bis „stimme voll zu“). In der Aufbereitung der Daten wurden die ersten 3 Grade zur Veranschaulichung grau („stimme nicht zu“) und die letzten 3 Grade blau („stimme zu“) hinterlegt.

Um die Antworten der Umfrageteilnehmenden in Bezug auf *innerhalb* und *außerhalb* der Palliativmedizin zu vergleichen, analysierten wir marginale mittlere Antwortwerte für paarweise Beobachtungen unter Verwendung der Scores 1 bis 6 [2]. Wir präsentieren Schätzungen der Unterschiede der marginalen Mittelwerte und entsprechende 95 %-Wald-Konfidenzintervalle.

### Ergebnisse

Versandt wurden laut BDA/DGAI-Geschäftsstelle 10.437 E‑Mails an Frauen (44,5 %) und 13.018 E‑Mails an Männer (55,5 %). Die Anzahl der Mitglieder betrug 15.129 (DGAI) und 20.167 (BDA) im November 2020. Es konnte keine Rücklaufquote bestimmt werden, denn einige Mitglieder hatten mehrere E‑Mail-Adressen angegeben, es bestanden Doppelmitgliedschaften und nicht alle Mitglieder hatten in den Erhalt von Rund-Mails eingewilligt.

Charakteristika der Teilnehmenden sind in Tab. 1 dargestellt. Von den *n* = 1384 TN gaben *n* = 1365 an, überwiegend als Ärzt:innen tätig zu sein, darunter 78 % im Krankenhaus, 63 % in der Anästhesie und 49 % in der Intensivmedizin Tätige. Der Anteil der Ärztinnen und Ärzte lag mit 46 % bzw. 54 % jeweils sehr nah an dem geschlechtsspezifischen Anteil der versandten E‑Mails. Die meisten Anästhesist:innen waren 41 bis 60 Jahre alt (58 %); das Durchschnittsalter der DGAI/BDA-Mitglieder lag bei 53 Jahren. Es werden nur die Daten derjenigen präsentiert, die angaben, überwiegend als Ärzt:innen tätig zu sein.

**Table 1:** 

**Alter [Jahre]**	**Teilnehmende, Umfrage (*n*** **[%])**
18–30	37 (3 %)
31–40	344 (25 %)
41–50	367 (27 %)
51–60	418 (31 %)
> 60	191 (14 %)
**Geschlecht**	
Frauen	621 (46 %)
Männer	731 (54 %)
**Berufserfahrung [Jahre]**	
≤ 10	254 (19 %)
11–20	395 (29 %)
> 20	710 (52 %)
**–**	**Ort der Tätigkeit aktuell** (Mehrfachnennung möglich)
Krankenhaus	1061 (78 %)
Anästhesie/OP	859 (63 %)
Intensivmedizin	669 (49 %)
Schmerzambulanz	186 (13,7 %)
Praxis	148 (11 %)
SAPV	99 (7 %)
AAPV	32 (2 %)
Hospiz	20 (1 %)
Altenpflege-Einrichtung	8 (< 1 %)
Anderer Bereich	201 (15 %)
**–**	**Umfang palliativmedizinischer Tätigkeit**
Vollständig/überwiegend	72 (5 %)
Teilweise	774 (57 %)
Gar nicht	514 (38 %)

Daten von Teilnehmenden, die angaben, Ärzt*innen zu sein. Die folgende Frage wurde jeweils übersprungen: Alter: 8, Geschlecht: 11, Berufserfahrung: 6, Tätigkeit aktuell: 4, Umfang palliativmedizinischer Tätigkeit: 5. Zwei Teilnehmende haben das Geschlecht als divers angegeben. Die Prozentsätze wurden anhand der Gesamtzahl der nichtfehlenden Beobachtungen berechnet

### Wahrnehmung des Umgangs mit M/O

**Figure 1:**
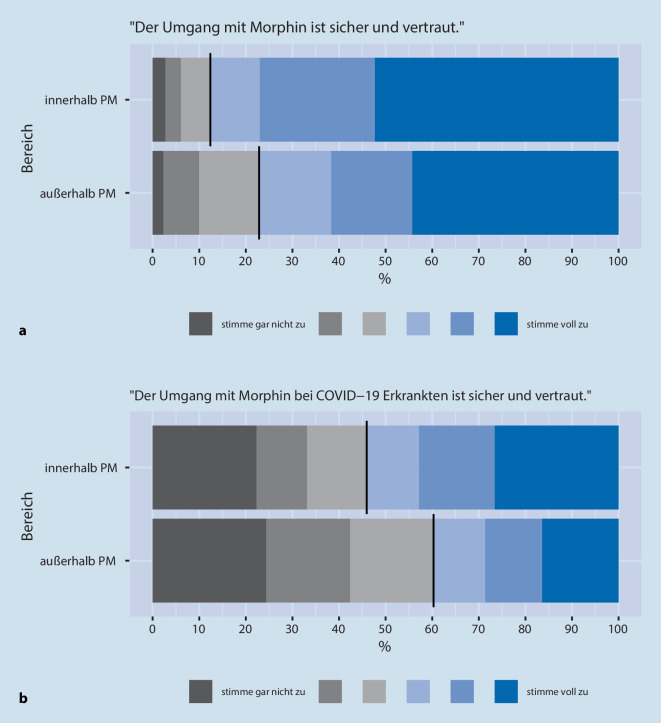

Die meisten Anästhesist:innen (88 %) schätzten den Umgang mit M/O für den Bereich *innerhalb* der Palliativmedizin als „sicher und vertraut“ ein, jedoch weniger (77 %) für den Bereich *außerhalb *der Palliativmedizin (Abb. 1a).

Bezogen auf die Frage nach einem sicheren und vertrauten Umgang mit M/O bei *COVID-19-Erkrankten*, wurde deutlich mehr Unsicherheit wahrgenommen: Nur 54 % stimmten für den Bereich *innerhalb* der Palliativmedizin zu, und nur 40 % für den Bereich *außerhalb* der Palliativmedizin (Abb. 1b). Intensivmedizinisch tätige Anästhesist:innen stimmten bei dieser Frage etwas häufiger zu (59 %, 46 %).

### Wahrnehmungen zur Klarheit der Regelung einer M/O-Gabe

**Figure 2:**
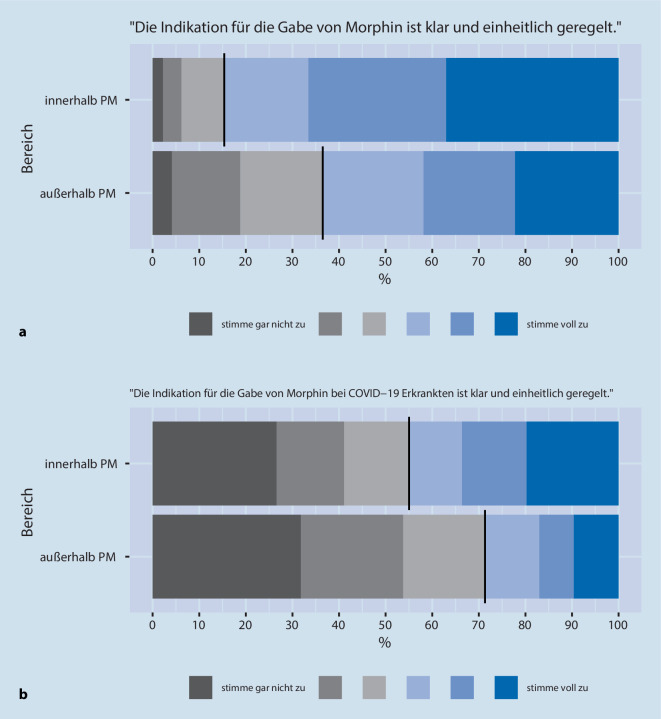

Eine klare Regelung für die Gabe von M/O sah ebenfalls der Großteil der Anästhesist:innen (85 %) für den Bereich *innerhalb* der Palliativmedizin, aber nur 63 % für den Bereich *außerhalb *der Palliativmedizin (Abb. 2a).

*Bezogen auf COVID-19-Erkrankte *beurteilten nur 45 % der Anästhesist:innen *innerhalb *der Palliativmedizin die Regelung zur M/O Gabe als klar und einheitlich geregelt, aber für den Bereich *außerhalb* der Palliativmedizin nur 29 % (Abb. 2b). Intensivmedizinisch tätige stimmten bei dieser Frage ebenfalls etwas häufiger zu (47 %, 33 %).

**Figure 3:**
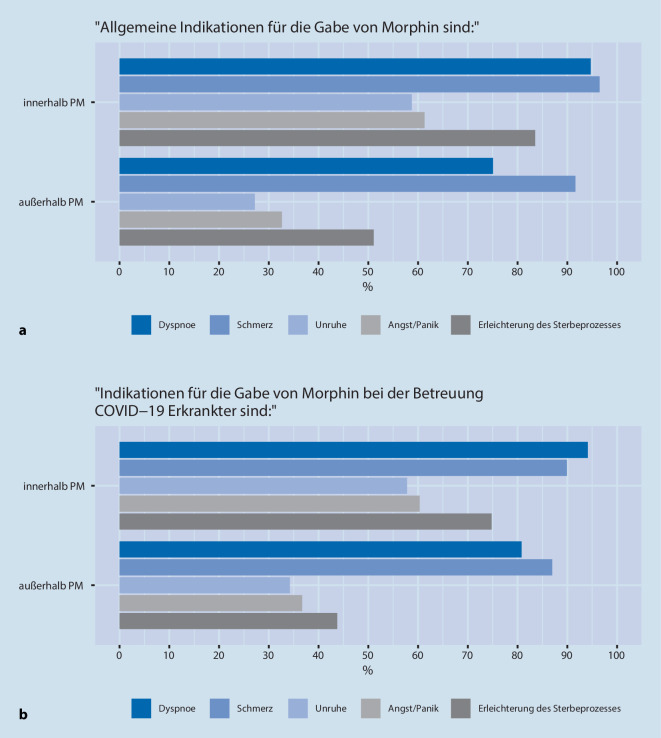

Die häufigsten allgemeinen Indikationen für die Gabe von M/O waren „Schmerz“ (96 %) und „Dyspnoe“ (95 %) *innerhalb *der Palliativmedizin (Abb. 3a), gefolgt von „Erleichterung des Sterbeprozesses“ (84 %), „Angst/Panik“ (61 %) und „Unruhe“ (59 %). Für den Bereich *außerhalb* der Palliativmedizin gaben die TN „Schmerz“ (92 %), „Dyspnoe“ (75 %), „Erleichterung des Sterbeprozesses“ (51 %), „Angst/Panik“ (33 %) und „Unruhe“ (27 %) als M/O Indikation an. Bei COVID-19-Erkrankten wurden die Indikationen zur Gabe von M/O ähnlich häufig angegeben. (Abb. 3a,b).

**Figure 4:**
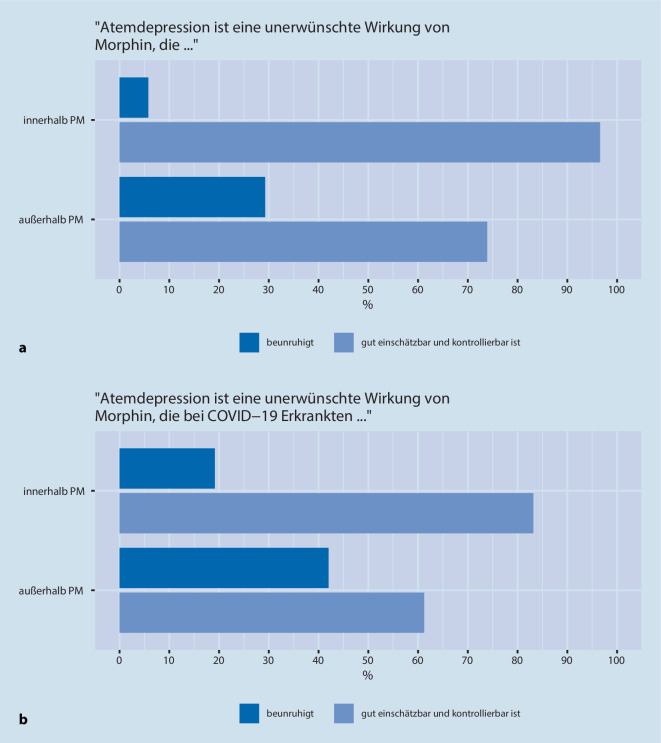

Ebenso beurteilten nahezu alle Anästhesist:innen (97 %) eine Atemdepression als unerwünschte Wirkung von M/O *innerhalb *der Palliativmedizin als „gut einschätzbar und kontrollierbar“. Jedoch hielten nur etwa 74 % der Opioidanwendenden diese Atemdepression *außerhalb *der Palliativmedizin für gut einschätzbar und kontrollierbar (Abb. 4a). Bezogen auf* COVID-19-Erkrankte, *wurde jeweils *innerhalb und außerhalb *der Palliativmedizin seltener angegeben, dass Atemdepression eine unerwünschte Wirkung von M/O ist, die gut einschätzbar und kontrollierbar ist (83 %/61 %) (Abb. 4b). Intensivmedizinisch tätige Anästhesist:innen gaben dies erneut diskret häufiger an (85 %, 66 %).

### Wahrnehmung zum Einsatz von M/O beim Sterben

**Figure 5:**
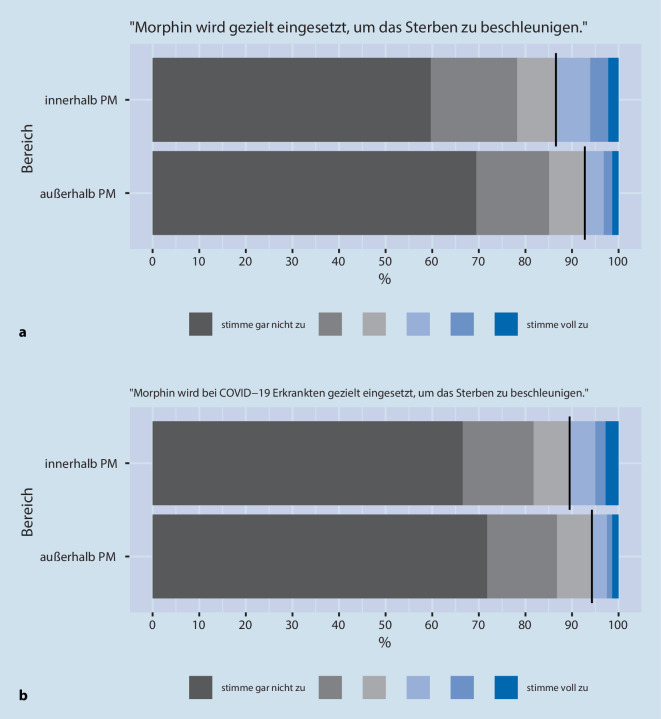

Die große Mehrheit der Anästhesist:innen negierte, dass M/O *innerhalb und außerhalb *der Palliativmedizin gezielt eingesetzt werde(n), um das Sterben zu beschleunigen (87 %/93 %). Hier fand sich kein Unterschied zu den intensivmedizinisch tätigen Anästhesist:innen. Die meisten der Teilnehmenden, die dieser Einschätzung zustimmten, gaben dies mit 13 % für den Bereich *innerhalb *der Palliativmedizin an (ohne COVID-19-Bezug) (Abb. 5a,b). Diese Teilnehmenden waren überwiegend männliche Ärzte (60 %), hatten über 20 Jahre Berufserfahrung (49 %) im medizinischen Bereich und waren im Krankenhaus (78 %), in Anästhesie/OP (63 %) sowie in der Intensivmedizin (52 %) tätig.

### Wahrnehmung zu bevorzugten Applikationswegen bei der Anwendung von M/O

Als bevorzugte Applikationswege von M/O wurden *innerhalb* der Palliativmedizin am häufigsten subkutan (76 %) und *außerhalb* der Palliativmedizin intravenös (78 %) angegeben. Die orale Gabe wurde etwa gleich häufig angeben – 66 % *innerhalb* und 62 % *außerhalb* der Palliativmedizin –, während eine transdermale Opioidgabe etwas seltener *außerhalb* als *innerhalb* der Palliativmedizin genannt wurde (38/47 %). Der größte Unterschied lag bei der mehr als doppelt so häufig genannten subkutanen Gabe *innerhalb* (76 %) vs. *außerhalb* der Palliativmedizin (32 %).

Zu der Aussage „Ich wünsche mir (mehr)“ gaben die Teilnehmenden an: „Einbindung eines palliativmedizinischen Konsilteams“ (85 %), „Teamkonferenzen“ (75 %), „Supervision“ (72 %) und „Lehre im Umgang mit Opioiden“ (69 %).

### Vergleich der Bereiche innerhalb vs. außerhalb der Palliativmedizin

Vergleicht man die Wahrnehmungen für die Bereiche *innerhalb* vs. *außerhalb* der Palliativmedizin, so stimmten die Anästhesist:innen eher für den Bereich *innerhalb* der Palliativmedizin zu, im Hinblick auf folgende Fragen: „Die Indikation für die Gabe von Morphin ist klar und einheitlich geregelt“ (Unterschied des Durchschnittswerts [*innerhalb* der Palliativmedizin – *außerhalb* der Palliativmedizin] 0,8; 95 %-Konfidenzintervall 0,7;0,9) und „Der Umgang mit Morphin ist sicher und vertraut“ (0,4; 0,3;0,5).

Diese Unterschiede fielen etwas schwächer bei den Fragen mit COVID-19-Bezug aus, aber weiterhin stimmten die Ärzt:innen eher für den Bereich *innerhalb* der Palliativmedizin zu, verglichen mit *außerhalb* der Palliativmedizin: „Die Indikation für die Gabe von Morphin bei COVID-19-Erkrankten ist klar und einheitlich geregelt“ (0,6; 0,5; 0,7), „Der Umgang mit Morphin bei COVID-19-Erkrankten ist sicher und vertraut“ (0,2; 0,1; 0,3).

Diese Tendenz zeigte sich sehr gering auch für die Fragen „Morphin wird gezielt eingesetzt, um das Sterben zu beschleunigen“ (0,3; 0,2; 0,4) und „Morphin wird bei COVID-19-Erkrankten gezielt eingesetzt, um das Sterben zu beschleunigen“ (0,3; 0,4;0,2).

## Diskussion

Anästhesist:innen nahmen teils große Unsicherheit im Umgang mit M/O wahr, insbesondere* außerhalb* der Palliativmedizin und bei der Betreuung COVID-19-Erkrankter. Leitlinien zur Symptomkontrolle von Dyspnoe sind heterogen, bezüglich einer Empfehlung zum Einsatz von Opioiden: Die COPD-Leitlinie der Deutschen Gesellschaft für Pneumologie warnt vor „bedeutsame(n), unerwünschte(n) Effekten, insbesondere der Atemdepression“, weswegen „der Einsatz auf wenige besonders beeinträchtigte Patienten mit schwerer Atemnot beschränkt“ sein solle [33]. Hingegen empfiehlt die allgemeine „S3-Leitlinie Palliativmedizin für Patienten mit einer nicht heilbaren Krebserkrankung“ (ohne COVID-19 Bezug) eindeutig Opioide zur Therapie von Schmerz und Dyspnoe (Atemnot) [24].

Die bis zum 16.05.2021 gültige, von 14 medizinischen Fachgesellschaften gemeinsam herausgegebene Version der „S3-Leitlinie – Empfehlungen zur stationären Therapie von Patienten mit COVID-19“ vermied die Nennung von Opioiden oder Benzodiazepinen zur Therapie von Dyspnoe [22]. Es wurde zwar festgestellt: „Der Palliativversorgung mit dem Ziel der optimalen Linderung von belastenden Symptomen wie Dyspnoe, Husten, Schwäche und Fieber, Angst, Panik, Unruhe und Delir kommt in diesen Situationen eine besondere Bedeutung zu“, konkrete Empfehlungen zur Symptomkontrolle mit Opioiden – wie sie in einer separaten Leitlinie der Palliativmedizin dargestellt waren [27] – fehlten jedoch. Eingang in die S3-Leitlinie zur stationären Therapie von Patient:innen mit COVID-19 fanden konkrete Dosierungsbeispiele erst während der dritten COVID-19-Welle am 17.05.2021 [23].

### Internationale Studien zu Opioiden bei Dyspnoe heterogen

Die Wirksamkeit von Opioiden bei Dyspnoe wurde insbesondere bei Patient:innen mit COPD, aber auch bei Herzversagen und Krebserkrankungen gezeigt [17, 21]: Hui und Bruera untersuchten in ihrem „evidenzbasiertem Review“ die pharmakologische Wirksamkeit speziell kurz wirksamer Opioide für die Linderung von „breathlessness“ [17]: Auf der Basis von 28 randomisierten klinischen Studien beurteilten sie die Evidenz insbesondere als gut, wenn kurz wirksame Opioide als Einzeldosis vor körperlicher Anstrengung verabreicht wurden. Johnson und Currow bestätigten retardierten Opioiden eine „moderate 1a-Evidenz“ [21]. Sie identifizierten die beste Evidenz für 10–30 mg retardiertes Morphin täglich „de novo“ bei opioidnaiven Patient:innen. Optimaler Nutzen wurde im Steady State gesehen.

Im Gegensatz dazu sah Vozoris in seinem „kritischen Review“ jedoch „bestenfalls eine leichte Verbesserung von Dyspnoe bei einer begrenzten Anzahl von Individuen mit COPD“ nach oraler Gabe von Opioiden [34]. Ebenso fanden Feliciano et al. in ihrer Metaanalyse und ihrem systematischen Review keine ausreichende Evidenz für den Einsatz von Opioiden zur Linderung von Breathlessness bei Patient:innen mit fortgeschrittener Krebserkrankung [14].

In Studien zur Sicherheit identifizierte Vozoris bei COPD-Patient:innen sogar „ein erhöhtes Risiko von respiratorisch-bedingten Exazerbationen, Hospitalisation und Tod in Verbindung mit Opioidgebrauch“ [34], während Johnson und Currow morphinbezogene Nebenwirkungen lediglich als „meist mild und selbstlimitierend, sobald die Substanz abgesetzt wird“ beschrieben [21]. Sie verglichen die Häufigkeit von ernsten Nebenwirkungen mit denen von Placebo. Die S3-Leitlinie Palliativmedizin betont darüber hinaus „Es gibt keinen Hinweis, dass eine lege artis durchgeführte Therapie der Atemnot mit Opioiden zu einer klinisch relevanten Atemdepression führt“[24].

Die Heterogenität der Leitlinien und internationalen Studien zur Dyspnoe könnte ein Grund für die wahrgenommene Unsicherheit im Umgang mit Opioiden in der hier präsentierten Umfrage sein. Darüber hinaus könnten Bedenken bezüglich Fehl- und Missbrauch von Opioiden [6] sowie bezüglich einer Interaktion von Morphin mit oralen Thrombozytenaggregationshemmern (P2Y_12_-Antagonisten) [26] zu weiterer Unsicherheit beitragen: So wurde der zuvor lang etablierte Standard der Morphingabe in den Guidelines der Europäischen Gesellschaft für Kardiologie zum akuten Koronarsyndrom ohne ST-Hebung 2015 mit einem Caveat versehen bzw. in der 2020-Version ganz entfernt [9, 26, 30].

Da Unsicherheiten im Umgang mit Opioiden seitens des medizinischen Fachpersonals in der Literatur mit einer unzureichenden Patientenversorgung assoziiert werden [6, 11, 13, 17], ist eine sichere, effiziente Symptomkontrolle bedeutend für eine adäquate Patientenversorgung. Im Falle der Notwendigkeit einer Triagierung sowie einer möglichen schnellen Verschlechterung bei der COVID-19-Erkrankung kommt somit der adäquaten Symptomkontrolle eine Schlüsselrolle zu.

### Individuelle Titration von Opioiden

Opioide sollten individuell und orientiert an der klinischen Wirkung titriert werden [17, 24, 31]. Potenziell unerwünschte Wirkungen wie Obstipation, Übelkeit, Sedierung, Atemdepression und ein nichtmedizinischer Gebrauch von Opioiden sollten bedacht werden.

Konkrete Dosierungsbeispiele zur Symptomkontrolle können in der Praxis hilfreich sein. Bei ca. zwei Dritteln der COVID-19-Patient:innen linderte eine subkutane, kontinuierliche Infusion mit einer Mediandosis von 15 mg Morphin und 10 mg Midazolam in den letzten 24 h ihres Lebens Dyspnoe und Unruhe [15, 16, 25]. In der S3-Leitlinie Palliativmedizin werden Opioide zur Therapie von Atemnot ausdrücklich empfohlen und 2,5–5 mg orales Morphin alle 4 h bzw. 1–2,5 mg Morphin alle 4 h s.c. als Startdosis bei opioidnaiven Patient:innen genannt [24].

### Spektrum der Anwendung von M/O

Die Behandlung von Schmerzen ist eine anerkannte Indikation für M/O, während die ebenfalls in der Umfrage genannten Indikationen Dyspnoe, Unruhe, Angst/Panik und Erleichterung des Sterbeprozesses dies nicht sind [5]. In Australien wurde ein Morphinpräparat erstmals zur symptomatischen Linderung chronischer Atemnot bei Patient:innen mit fortgeschrittenen und terminalen Erkrankungen zugelassen [4, 21]. Während Schmerz und Dyspnoe als die häufigsten Indikationen in unserer Umfrage genannt wurden, nahmen die Teilnehmenden insbesondere Unruhe sowie Angst/Panik nur halb so oft für den Bereich *außerhalb* der Palliativmedizin wahr.

Die Abfrage der Indikationen in dieser Umfrage (Abb. 3a,b) basierte auf den typischen Fragestellungen und Assoziationen zum Umgang mit M/O, die wir zuvor in einer qualitativen Erhebung im Rahmen von Interviews und auf Grundlage der Literatur identifizierten (nicht publiziert, s. Abschn. „Material und Methoden“). So entstand auch die Nennung einer „Erleichterung des Sterbeprozesses“ durch M/O, die an das Konzept des „dying well“ erinnert, das von der Begründerin der Hospizbewegung, Cicely Saunders, beschrieben wurde [32] und deutlich von einer aktiven Sterbehilfe abzugrenzen ist.

In der Literatur finden sich weitere Hinweise für einen sinnhaften Einsatz über die klassischen Indikationen hinaus: z. B. bei Agitation, die ein Zeichen für Schmerzen sein kann [18]. Auch wird diskutiert, ob M/O bei einem Teil der COVID-19-Erkrankten Stress reduzieren kann [28]. Hier sollten im klinischen Setting ergänzende und alternative Konzepte wie Benzodiazepine zur Behandlung von Dyspnoe und Unruhe sowie auch nichtpharmakologische Interventionen erörtert werden, wie auch von Rosenbruch et al. empfohlen [31].

Die subkutane Gabe von Opioiden scheint eine „Domäne“ der Palliativmedizin zu sein, da sie mit 76 % mehr als doppelt so häufig für den Bereich *innerhalb* der Palliativmedizin berichtet wurde. Dieses Phänomen ist *innerhalb* der Palliativmedizin bekannt und könnte als unkompliziertes, gut zu praktizierendes Modell für andere Fachbereiche dienen [15, 20].

### Beschleunigen M/O das Sterben?

Ein deutlicher Warnhinweis war die Bewertung bei 13 % der Ärzt:innen (*n* = 169), dass M/O *innerhalb *der Palliativmedizin gezielt eingesetzt werden, um das Sterben zu beschleunigen (Abb. 5). Allerdings kann dabei nicht zwischen einer Intention der Beschleunigung im Sinne von aktiver Sterbehilfe oder nur einer Inkaufnahme eines potenziell schnelleren Versterbens im Rahmen der beabsichtigten verbesserten Symptomkontrolle differenziert werden. Die Inkaufnahme nach dem Prinzip des „doppelten Effekts“ wäre in der ethischen Bewertung akzeptabel, wenn M/O alternativlos zur Symptomkontrolle eingesetzt werden, dabei aber auch das Risiko akzeptiert wird, dass dadurch zu einem früheren Versterben beigetragen werden könnte [7, 8]. Eine Opioidbehandlung mit primär beabsichtigter Beschleunigung des Sterbens hingegen wäre juristisch in Deutschland nicht akzeptabel und könnte einem Tötungsdelikt entsprechen und damit eine strafbare Handlung nach §216 StGB darstellen, wenn die Maßnahme als „Tötung auf Verlangen“ durchgeführt wird (der Patient muss den Arzt aufgefordert haben). Eine Gabe eines Opioids ohne „Verlangen“ des Patienten mit dem Ziel, das Sterben zu beschleunigen, kann strafrechtlich als Mord (§211) oder Totschlag (§212) gewertet werden.

Allerdings ist bei einer sachgerechten und individuell angepassten Opioid-Titration bei sterbenden Menschen mit Schmerzen nicht zu erwarten, dass sie vorzeitig an einer solchen Opioidgabe versterben: Eine retrospektive Studie stationär eingewiesener Patient:innen, die eine reine Symptomkontrolle und Änderung der Opioiddosis erhielten, zeigte keinen signifikanten Unterschied bezüglich der Zeit bis zum Versterben in der Niedrigdosis- vs. Hochdosisgruppe [1]. Das Einbeziehen einer palliativmedizinischen Konsultation hingegen erhöhte die Überlebenszeit sogar signifikant.

### Rollen der Anästhesiologie und der Palliativmedizin

Historisch gesehen hat die Anästhesie eine Schlüsselrolle in deutschen Krankenhäusern bezüglich der Entwicklung und Institutionalisierung von Schmerz- und Palliativmedizin [12]. Der Fähigkeit zur interdisziplinären und schnittstellenübergreifenden Arbeitsweise von Anästhesist:innen wird die Weiterentwicklung der Palliativmedizin zugeschrieben [12]. Fachpolitische Prozesse in der Anästhesiologie integrierten die Palliativmedizin als „fünfte Säule der Anästhesiologie“ durch Hinzunahme eines „P“ in das Akronym „AINSP“ [12, 19].

Die internationale Literatur sieht in der Palliativmedizin eine „essenzielle Antwort auf die COVID-19-Pandemie“, da spezialisierte palliativmedizinische Dienste in der Pandemie flexibel und bedarfsangepasst reagieren konnten und so eine wichtige Rolle in der Versorgung und Symptomkontrolle von COVID-19-Erkrankten spielten [10, 25]. Neben lange bestehenden Forderungen nach einer frühen Integration von Palliativmedizin zur Verbesserung der medizinischen Versorgung [6] fordern Fadul et al. explizit eine Integration von Palliativmedizin auch in die COVID-19-Pandemie-Planung [13]. So wurde – neben Einbindung in Entscheidungsalgorithmen – eine Begleitung des medizinischen Personals im Hinblick auf Erhaltung der Arbeitsfähigkeit vor dem Hintergrund möglicher psychisch belastender Entscheidungen unterstützt.

Die „CHARTA zur Betreuung schwerstkranker und sterbender Menschen in Deutschland“ benennt bereits im Leitsatz 2: „Wir werden uns dafür einsetzen, dass Versorgungsstrukturen vernetzt und bedarfsgerecht für Menschen jeden Alters und mit den verschiedensten Erkrankungen mit hoher Qualität so weiterentwickelt werden, dass alle Betroffenen Zugang dazu erhalten“ [3, S. 19]. Diese Leitsätze bildeten die Grundlagen für notwendige Prozesse zur verbesserten Versorgung von palliativmedizinischen Patient:innen.

### Limitationen

Die Umfrage fand statt zu Beginn der zweiten COVID-19-„Welle“ in Deutschland. Einige Teilnehmende erwähnten, dass sie noch keine COVID-19-Erkrankten behandelt hätten und daher die Fragen mit COVID-19-Bezug übersprungen hätten.

Es konnte keine Rücklaufquote bestimmt werden (s. Abschn. „Ergebnisse“). Der Vergleich der Teilnehmenden mit den Mitgliederdaten zeigte eine ähnliche Struktur von Geschlechter- und Altersverteilung, sodass hier zumindest eine Ähnlichkeit angenommen werden darf. Dass 814 Mitglieder (71 %) nicht zustimmten, die Gabe von M/O bei COVID-19-Erkrankten *außerhalb* der Palliativmedizin als klar und einheitlich geregelt wahrzunehmen, dürfte Anlass genug sein, diese Unsicherheit in Zukunft besser verstehen zu wollen.

Von einigen Teilnehmenden wurde trotz erläuternder Einführung im Fragebogen kritisiert, dass nicht immer klar war, um welche Situationen es sich in den gestellten Fragen handelte. So wurde geäußert, dass die Unterscheidung zwischen *innerhalb* und *außerhalb* der Palliativmedizin teils als unscharf wahrgenommen wurde. Der Bereich „*außerhalb* der Palliativmedizin“ bezieht sich auf sämtliche andere Fachbereiche und kann sehr heterogen verstanden worden sein. Zusammengefasst sollte die Bereichszuordnung als grobe Einschätzung eingeordnet werden.

Morphin wurde im Originalfragebogen stellvertretend für die Gruppe der Opioide genannt und initial entsprechend erläutert. Diese Festlegung wurde von einzelnen Teilnehmenden unterschiedlich wahrgenommen, da es an einigen Kliniken teils etablierte Konzepte mit anderen Opioiden gibt. Wir hatten Morphin gewählt, da es unserer Einschätzung nach das meistangewandte Opioid in der Klinik sowie in der hier referierten internationalen Literatur ist. Dadurch sollte für die Umfrage Teilnehmenden ein konkreter Bezug zu Situationen im Klinik‑/Praxisalltag leichter hergestellt werden können.

## Fazit für die Praxis

- Die wahrgenommene, teils deutliche Unsicherheit im Umgang mit M/O unter Anästhesist:innen sollte ernst genommen werden. Dies ist ein klares Signal für die Notwendigkeit der Erstellung einheitlicher, interdisziplinärer Leitlinien mit Dosierungsempfehlungen von Opioiden.
- Die häufig wahrgenommene subkutane Gabe von Opioiden *innerhalb* der Palliativmedizin sollte aufgrund der guten Praktikabilität als Inspiration zur Symptomkontrolle für Fachkliniken außerhalb der Palliativmedizin dienen. Arzneimittelzulassungen könnten ggf. bezüglich einer Zulassungserweiterung zur subkutanen Gabe geprüft werden.
- Mehr Ausbildung und Lehre sowie die Unterstützung durch ein palliativmedizinisches Konsilteam könnten Unsicherheiten im Umgang mit Opioiden adressieren. Kollegiale Unterstützung bei der Betreuung COVID-19-Erkrankter könnte ein teils angespanntes Arbeitsumfeld während der Pandemie entlasten.
